# The Portrait of Cyberchondria—A Cross-Sectional Online Study on Factors Related to Health Anxiety and Cyberchondria in Polish Population during SARS-CoV-2 Pandemic

**DOI:** 10.3390/ijerph19074347

**Published:** 2022-04-05

**Authors:** Marta Ciułkowicz, Błażej Misiak, Dorota Szcześniak, Jolanta Grzebieluch, Julian Maciaszek, Joanna Rymaszewska

**Affiliations:** 1 Department of Psychiatry, Wroclaw Medical University, 50-367 Wroclaw, Poland; blazej.misiak@umw.edu.pl (B.M.); dorota.szczesniak@umw.edu.pl (D.S.); julian.maciaszek@umw.edu.pl (J.M.); joanna.rymaszewska@umw.edu.pl (J.R.); 2 Department of Population Health, Wroclaw Medical University, 50-367 Wroclaw, Poland; jolanta.grzebieluch@umw.edu.pl

**Keywords:** cyberchondria, pandemic, health anxiety

## Abstract

The SARS-CoV-2 pandemic has served as a magnifying glass for cyberchondria, while the internet emerged as one of the main sources of medical information and support. The core ambition of this study was to estimate the level of cyberchondria and describe the socio-demographic, clinical and pandemic-related factors affecting its severity amid the SARS-CoV-2 pandemic. A cross-sectional study was performed between 16 May 2020 and 29 December 2020 in Poland within a sample of 538 adult internet users. The online survey tool included a Polish adaptation of the Cyberchondria Severity Scale (CSS-PL) and the Short Health Anxiety Inventory (SHAI), complemented with a set of questions covering sociodemographic, clinical and pandemic-related factors. Participants were clustered according to severity of health anxiety and cyberchondria symptoms. The performed binary logistic regression indicated professional inactivity, having a chronic mental disorder and subjectively limited access to healthcare due to COVID-19 to be key determinants of severe health anxiety and cyberchondria. Cyberchondria might be a remarkable public health issue as large proportion of respondents from the analyzed sample population of internet users met the criteria for severe symptoms. Key determinants of intense cyberchondria corresponded with employment stability, mental resilience and accessibility of healthcare services, which could be greatly challenged amid the pandemic.

## 1. Introduction

Previous epidemics and pandemics of viral infections, such as SARS [1], AH1N1 [2], or Ebola [3], provided fertile ground for developing anxiety, which also emerged to be a common issue during the COVID-19 outbreak [4,5,6]. Health anxiety is a continuous construct [7] ranging in intensity from almost none to severe. While a certain alertness towards one’s health status could be understood as an advantageous evolutionary mechanism, excessive health anxiety considerably disrupts daily functioning [8] and facilitates uncontrolled searching for medical data [9]. The intensified use of the internet in this context can be interpreted as a safety-seeking behavior [10], to subjectively estimate the probability of the illness and then prospectively dismiss such a scenario, whereas in reality, pessimistic information [11] or exceptionally dramatic media coverage of health-related issues [12] may worsen the subjective distress. This interplay could be explained by the cognitive-behavioral model of health anxiety [13], elaborating on Beck’s cognitive theory of psychopathology [14]. According to the model, distorted beliefs and erroneous schemas result in behavioral, emotional, and physical reactions. For example, habitually perceiving mild bodily symptoms as related to a serious medical condition may lead to an amplification of these sensations. Jokic-Begic et al. [15] claim that searching the internet for medical content intensified during the pandemic. In a medical milieu, a certain behavioral pattern (repeatedly and/or excessively reviewing medical content on the internet) that escalates the emotional burden (mainly health anxiety) is called “*cyberchondria*” [16,17]. Thus, individuals with cyberchondria experience amplified anxiety instead of support and relief in the course of searching for virtual content. Importantly, the aforementioned searches are compulsive and persistent in nature [18]. The term was probably coined by Ann Carrns in the pages of the Wall Street Journal in 1999 as a reaction to anticipated threats related to the advent of the internet. Jungman et al. [19] deduce, based on work done by Williams [20] and Witthoft et al. [21], that the vicious circle in cyberchondria roots in predisposing factors and is sustained by negative reinforcement. A systematic review by Vismara et al. [18] summarizes that a growing body of research confirms the relationship of cyberchondria with health anxiety, hypochondriasis, obsessive compulsive disorder, and problematic usage of the internet. The supposed close link with hypochondriasis was emphasized by using the name “*cyberchondriasis*” by some authors [22,23,24]. Up until now, the course of cyberchondria is said to be modified by personality characteristics such as optimism and neuroticism [25], low self-esteem [26], anxiety sensitivity [27,28], particular meta-cognitive beliefs [29], pain catastrophizing [30] and intolerance of uncertainty [28,31,32]. Although cyberchondria uniquely affects functional impairment and alters service use when compared to health anxiety alone [33] and comes with considerable cost and burden [24], it is not regarded a separate construct. Hence, it has been included in neither the Diagnostic and Statistical Manual of Mental Disorders (DSM) nor the International Classification of Diseases (ICD). Conversely, a suitable definition can be found in the Oxford English Dictionary: “*a person who (obsessively) researches health information on the Internet, typically to find a disease matching particular (real or imagined) symptoms*”. Additionally, it was announced to be a finalist in the “2008 Word of the Year” held by Webster’s New World [34]. Growing recognition of cyberchondria in the non-medical literature contrasts with a major knowledge deficit concerning vulnerability factors that contribute to higher levels of anxiety during internet searches and their compulsive backlash [18]. Notably, while high baseline health anxiety is not the essential factor for cyberchondria development [35], resources exploring individual background characteristics remain scarce and often contradictory [18]. What is certain is that prolonged and recurrent distress in the face of menace may facilitate a cycle of distress [36,37] and lead to further information seeking concerning a stressful event [38]. The phenomenon of rapid and massive pandemic-related information production was observed and announced by the World Health Organization as the “*infodemic*”, co-existing with the actual biological threat [39]. Undeniably, the internet is critical for timely and constant sharing of recommendations and updates to enhance preparedness and adequate response to the SARS-CoV-2 pandemic by healthcare professionals and governments. At the same time, the penetration of solid information could have been uneven as the general population of Internet users was simultaneously exposed to immoderate and emotionally driven coverage of the pandemic curated by private users on social media [40]. Eichenberg et al. [41], investigating patterns of online health resource use, observed that people with hypochondria are more eager to search online for health-related content and employ more services available on the internet. Under such circumstances, their e-health literacy could be seriously challenged [38]. For example, McDonnell et al. [42] observed media-induced anxiety towards H1N1 influenza within a relatively unimpacted community that increased of visits to the Emergency Department comparable to rates expected in affected regions.

In summarizing, cyberchondria is a relatively novel concept, observed to be potentially distinct from the anxiety disorder spectrum. From the perspective of a pandemic-related mental health disaster [43] and massive misinformation regarding the threat, it seems crucial to pay attention to all the phenomena that could be psychopathological. The study aimed to assess the level of cyberchondria and describe sociodemographic, clinical, and pandemic related factors affecting its severity amid the current epidemiological crisis.

## 2. Materials and Methods

### 2.1. Participants

Data were collected through a cross-sectional online survey, made available to the participants between 16 May 2020 and 29 December 2020, in Poland. The snowball sampling method was applied to recruit adult representatives of the Polish population of internet users. Researchers nominated their colleagues, friends, families and followers on social media to distribute the survey within internet users eligible for the study. The inclusion criteria involved adult age, computer literacy and access to the internet. The Computer Assisted Web Interviews (CAWI) method was employed [44]. Respondents were informed about the voluntary, confidential, and anonymous character of the study. This information was provided at the very beginning of the questionnaire. Submitting a filled survey designated that respondent was familiar with the study’s goal, description, reached adulthood as well as agreed to the terms of participation in the research. Data analysis was limited to completed questionnaires. The Ethics Committee at Wroclaw Medical University (Poland) approved the study protocol (approval number: 286/2020). The study was performed in agreement with the principles of the Declaration of Helsinki. The paper structure was based on STROBE statements for reporting cross-sectional studies [45]. 

### 2.2. Measures

*The Polish adaptation of Cyberchondria Severity Scale (CSS-PL*) [26] based on the work by McElroy et al. [17] is a 33-item scale that enables complex assessment of cyberchondria. Items are arranged in 5 sub scales: compulsion (item 3, 6, 8, 12, 14, 17, 24, 25), distress (item 5, 7, 10, 20, 22, 23, 29, 31), excessiveness (item 1, 2, 11, 13, 18, 19, 21, 30), reassurance (item 4, 15, 16, 26, 27, 32) and mistrust of medical professional (item 9, 28, 33). The answers are ranged on 5-point Likert scale (1-never, 2-rarely, 3-sometimes, 4-often, 5-always). The higher the score, the more intense the experienced symptoms. Cronbach’s alpha for the Polish adaptation was consistent with the original version [17] and ranged between 0.75 and 0.95. In our research, it was estimated at 0.90.

*The Polish adaptation of Short Health Anxiety Inventory* performed by by Kocjan [46] is a 16-item self-administered register based on The Short Health Anxiety Inventory (SHAI) by Salkovskis [47]. It comprises 18 items exploring two elements of hypochondriasis: illness likelihood (IL) and negative consequences of an illness (NC). However, the general score can also be considered and understood as a summation of the points. Each item is comprised of four statements related to the last 6 months. Participants were asked to choose one as an equivalent to a 4-point Likert scale, where the first answer suggested *no* symptoms (0), second *mild symptoms* (1), third *severe symptoms* (2) and fourth *very severe symptoms* of clinical hypochondriasis. Cronbach’s alfa of the Polish adaptation was described as excellent as it exceeded 0.90 [46]. It was evaluated to be 0.92 in the current study.

*The questionnaire on socio-demographic, clinical and pandemic-related factors* consisted of 16 questions developed based on a literature review. Fourteen questions were closed and allowed participants to choose one answer describing them the most accurately. Two open questions were designed to record the number of contacts with both mental and non-mental health services. The questions were not piloted. They covered variables such as age, gender, education, working status including remote work, place of residence, number of household members, engagement in social meetings or time spend on the internet during the day. Respondents disclosed if they were living with any chronic physical or mental illnesses and estimated how many times they used mental and somatic health services during last month including online consultations. Additionally, data regarding probable pandemic-related life circumstances such as work loss or trust in online COVID-themed contents were recorded. The questions on the pandemic were used to obtain the subjective assessments of the respondents.

### 2.3. Data Analysis

Statistical analysis was performed using the Statistical Package for Social Sciences, version 20 (SPSS Inc., Chicago, IL, USA) [48]. As mistrust of medical professionals factor of CSS poorly correlated with the global cyberchondria, it was regarded to be only theoretically associated with the phenomenon of cyberchondria [10,26,45,46]. With this in mind, it was not analyzed. Participants were divided into two groups based on the CSS and SHAI scores and cut-off points established using the k-means cluster analysis. Individuals with primary and secondary educational background were labelled as *lower educated* and analyzed together due to their very limited representation in our study. Between-groups differences in continuous variables were tested using the Mann–Whitney U test due to non-normal distribution (the Kolmogorov–Smirnov test). The chi-square test was used to compare distribution of categorical variables. Significant associations in bivariate tests were further explored using the binary logistic regression analysis. The group status employed according to the k-means cluster analysis was included as the dependent variable. The level of significance was set at *p* < 0.05.

## 3. Results

**Figure 1:**
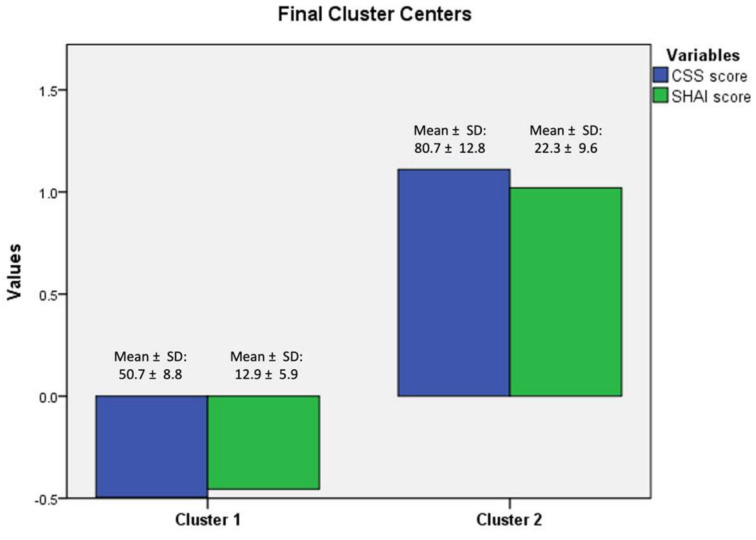
Results of k-means cluster analysis.

Out analysis revealed two prominent clusters (Figure 1). The first cluster included 372 individuals with low center scores of health anxiety and cyberchondria, while the second cluster of 166 people was characterized by high center scores in both measures. Means, standard deviation values and ranges regarding CSS-PL and SHAI total outcomes within particular clusters are presented in Table 1. 

The majority of the respondents within emerged clusters were well-educated women (*n* = 438, 81.4%), co-habiting with at least one person (*n* = 482, 89.6%) in urban environments (*n* = 468, 87.0%). The mean age of all respondents in our study sample was 36.7 ± 12.5 years. Table 2 shows bivariate comparisons of individuals representing both clusters. Being professionally (*p* < 0.05) and socially active in the preceding month (*p* < 0.05) were significantly more frequent within individuals with low levels of cyberchondria and health anxiety. Participants clustered as experiencing severe cyberchondria and health anxiety reported chronic mental comorbidity significantly more often (*p* < 0.001). Similarly, the use of both psychiatric (*p* < 0.001) and non-psychiatric (*p* < 0.05) services in that group was relevantly more frequent. Even though study participants in Cluster 2 were significantly keener on using online consultations with medical professionals in the previous month (*p* < 0.05), they also self-reported limited access to medical care due to the COVID-19 pandemic (*p* < 0.001). No significant differences between clusters were found in age, gender, education, place of residence, number of household members, remote work, job loss and trust in online contents covering COVID-19 and chronic somatic comorbidity. The results of the binary logistic regression can be found in Table 3. The key determinants of high CSS and SHAI total scores were professional inactivity (B = 0.535, *p* < 0.05), having a chronic mental disorder (B = 0.933, *p* = 0.001) and subjectively limited access to care due to COVID-19 (B = 0.781, *p* < 0.05). At the same time, the number of contacts with psychiatric and somatic medical care units, the use of online consultations in the previous month along with involvement in social gatherings did not significantly determine higher scores of cyberchondria and health anxiety.

## 4. Discussion

Based on the available data, it could be assumed that 86.8% of the Polish population were internet users in 2020, when adult population was about 31 million [49]. Hence, our sample represents approximately 538 of 27 million adult internet users in Poland at that time [50]. In our sample, 166 of 538 (30.9%) internet users were clustered as those experiencing high level of cyberchondria symptoms as well as health anxiety. To the best of our knowledge, to date this is the only research aimed at analyzing the phenomenon of cyberchondria in the general population of internet users during the pandemic. Existing data refer to a narrow group of dental students surveyed by Shailaja et al. [51]. According to this study, as many as 98.7% of 404 answerers were moderately or severely affected by any of cyberchondria symptoms, amid the epidemiological crisis. Nevertheless, the authors did not refer to the CSS total scores in the sample. Pre-pandemic research on cyberchondria was carried out in various groups of respondents and indicated discrepancies in the severity of this phenomenon. Aulia et al. [52], stimulated by the idea of the “medical student syndrome”, examined 162 first-year students in Indonesia, and concluded that 37.65% present symptoms of cyberchondria. Seven percent of participants scored positive for cyberchondria according to the CSS threshold estimated by the ROC curve analysis. Wijesinghe et al. [53], in turn, focused on outpatients from two general hospitals in Sri Lanka and estimated the prevalence of distinct symptoms of cyberchondria at 16.3%. Akhtar et al. [54] analyzing a group of graduates aged at least 35 years, with no chronic medical condition, found that 24.3% of respondents experienced acute symptoms of cyberchondria, while 50.0% reported moderate symptoms. Makarla et al. [55] found 55.6% of the surveyed technology sector workers to potentially have prominent cyberchondria symptoms, using a cluster approach. Moreover, White et al. [56] observed that 38.4% of the representatives of a general population sample reported a progression from low baseline health anxiety to more severe health anxiety, while searching the web for over 11 months. Our observations seem consistent with the pre-COVID body of research. However, the obtained results could be associated with the period of data collection, since at the end of April 2020, strict restrictions connected to the first national lockdown were gradually lifted in Poland. Consequently, Polish citizens partly regained their flexibility and freedom to, for example engage in recreational activities within common spaces, if personal protective equipment was used. This could have enhanced their sense of control and given them hope for overcoming the health crisis. Although previous research has suggested the potential role of sociodemographic factors such as age, gender, and education in cyberchondria, our results do not corroborate these findings. These variables did not differ across both clusters and were not found to be determinants of intense health anxiety as well as cyberchondria. Thus, they did not satisfactorily explain our outcomes. It could be hypothesized that intrapsychic factors play a greater role in cyberchondria intensity. The exploration of this area may be of utmost importance during the pandemic as such a crisis could blend the boundaries between internal and external menaces. When such boundaries are vague, the external threats related to the pandemic are additionally powered by unconscious internal vulnerabilities. In consequence, generated emotional tension may find an outlet for example through certain behavior or attitudes towards people, objects or situations [57,58]. Nevertheless, personality traits were not examined in this study. Taking matters further, attention should also be paid to interpersonal factors such as social networks. We noticed that respondents with high levels of health anxiety and cyberchondria were less eager to engage in social meetings. This is somewhat consistent with the observation made by Farooq et al. [40] that experiencing cyberchondria during pandemic may facilitate the intention to self-isolate. Simultaneously, no significant difference between the clusters was found regarding remote work which could be potentially appreciated by people with health anxiety and cyberchondria amid the COVID-19 pandemic. Professional inactivity, in turn, determined severe health anxiety and cyberchondria symptoms. It could be presumed that unstructured daily routines may favor unrestricted internet searches in order to find free medical information and support. Such searches may be hypothetically fueled by symptoms of anxiety, depression, and somatization that are more prevalent in that group when compared to working individuals [59]. Nonetheless, job loss due to the pandemic did not significantly vary between clusters. Furthermore, Bajcar et al. [26] suggest that adopting measures to prevent cyberchondria symptoms may reduce a risk of developing various disorders. This is somewhat consistent with our results, which demonstrated that living with a chronic mental disorder was more prevalent in the cluster characterized by high health anxiety and high cyberchondria as well as was found to be a significant determinant of more severe symptoms. Besides, the use of psychiatric and non-psychiatric offline consultations was significantly more prevalent in the cluster characterized by high levels of health anxiety and cyberchondria in the current research, while at the same time, our results suggest that individuals with severe health anxiety and cyberchondria were less eager to use online consultations. Tanis et al. [60] noticed that health anxiety is positively related to searching for medical information on the internet and individuals experiencing such symptom are satisfied with medical consultations to a lesser extent. Likewise, individuals with cyberchondria could have a negative attitude toward medical staff and not consider online health-related data as a proper substitute for a professional consultation [61]. These observations could at least partly explain the reluctant approach towards online counselling within the high health anxiety and high cyberchondria cluster in our study, along with self-reported restrained access to medical care as that was largely moved to the virtual space due the pandemic. Conversely, Eichenberg et al. [41] concluded that such behavior is not a consequence of limited access to offline services but rather tendency to double-check received information. 

### Limitations

The results of this research must be considered in light of several limitations. The vague definition of cyberchondria and the lack of a fixed cut-off score of the variants of CSS may hinder the proper assessment of the severity of this phenomenon and comparability across the existing literature. The cross-sectional and self-report design of the study prevents us from confirming casual relations between the analyzed variables. This study was inspired by the first wave pandemic and a related surge of internet traffic. Notably, no data were collected in pre-pandemic period as well as no follow-up was performed. Caution should be used not to generalize results without contemplating mentioned circumstances. Replication using longitudinal and experimental methodology is necessary. It should also be noted, that our sample might be characterized by low representativeness as we did not control for the initial number of individuals approached for participation. Consequently, the response rate and extent and reasons of non-participation were not recorded. For similar reasons, the response rate was not recorded. On one hand, online data collection surveys are suitable for large and diverse samples, on the other, response rates in web surveys are generally low, which may introduce high non-response errors [44]. Similarly, an inadequate representation of individuals with basic or secondary education made comparisons between those two education levels inaccurate. At the same time, data concerning respondents’ professions were not collected. Moreover, the vast majority of our respondents were well-educated women. As gender may imply different psychosocial consequences of the COVID-19 pandemic [62,63], it would be interesting to elaborate if the burden of traditional gender roles may be an independent determinant of cyberchondria intensity. Despite considering the physical and mental co-morbidity of the study participants, we did not analyze individual medical records. Likewise, we did not control for trait anxiety. A greater variety in age ranges, namely incorporating representation of both young adults and seniors could complement the investigation of an interplay between sociodemographic variables and cyberchondria symptoms severity. Finally, any research during the pandemic should be interpreted in light of local pandemic-related restrictions. It could be hypothesized that at the earliest stages of strict national lockdowns, the severity of symptoms among vulnerable populations could have been even greater.

## 5. Conclusions

The present study indicates that a large proportion of the analyzed sample might experience cyberchondria symptoms. This phenomenon might be associated with occupational inactivity, the diagnosis of a chronic mental disorder and restricted access to medical care due to the COVID-19 pandemic. As employment stability, mental resilience and organization of healthcare services are seriously challenged amid the current crisis, these problem areas should be addressed in both clinical practice and future research. Providing patients with information on how to effectively obtain proper medical and social support could possibly alleviate the symptoms of cyberchondria and improve the therapeutic relationship. Moreover, further research in this field should explore interpersonal as well as intrapersonal factors, including personality traits, which determine the severity of cyberchondria symptoms.

## Figures and Tables

**Table 1:** General characteristics of the emerged clusters based on CSS-PL and SHAI total scores.

	Cluster 1	Cluster 2
	mean ± SD (range)	mean ± SD (range)
CSS-PL	50.7 ± 8.8 (30–68)	80.7 ± 12.8 (63–129)
SHAI	12.1 ± 5.9 (0–35)	22.3 ± 9.6 (2–51)

**Table 2:** General characteristics of the sample with respect to clusters of the CSS and SHAI scores (*n* = 538).

	Total Sample *n* = 538	Cluster 1 (Low Scores of CSS-PL and SHAI)		Cluster 2 (High Scores of CSS-PL and SHAI)		*p*
	Mean ± SD or *n* (%)	*n*	Mean ± SD or *n* (%)	*n*	Mean ± SD or *n* (%)	
Age, years	36.7 ± 12.5	372	36.7 ± 12.8	166	36.6 ± 11.9	0.682
Gender, males	100 (18.6)	372	74 (19.9)	166	26 (15.7)	0.244
Education, higher	422 (78.4)	372	297 (79.8)	166	125 (75.3)	0.237
Active working status, yes	397 (73.8)	372	290 (78.0)	166	107 (64.5)	**0.001**
Place of residence, urban	468 (87.0)	372	320 (86.0)	166	148 (89.1)	0.336
Number of other household members > 1	482 (89.6)	372	330 (88.7)	166	152 (91.6)	0.316
Remote work, yes	245 (45.5)	369	171 (46.3)	164	74 (45.1)	0.794
The loss of work due to the COVID-19 pandemic, yes	22 (4.1)	372	13 (3.5)	166	9 (5.4)	0.579
Trust in online contents about the COVID-19, yes	56 (10.4)	372	33 (8.9)	166	23 (13.9)	0.176
Involvement in social meetings during the preceding month, yes	316 (58.7)	372	230 (61.8)	166	86 (51.8)	**0.029**
Chronic somatic diseases, yes	97 (18.0)	372	66 (17.7)	166	31 (18.7)	0.795
Chronic mental disorders, yes	94 (17.5)	372	42 (11.3)	166	52 (31.3)	**<0.001**
The use of online consultations with medical professionals in the preceding month, yes	206 (38.3)	370	125 (33.8)	166	81 (48.8)	**0.001**
The number of contacts with medical care units in the preceding month (without mental health services)	1.0 ± 1.6	372	0.9 ± 1.6	166	1.1 ± 1.6	**0.005**
The number of contacts with mental health services in the preceding month	0.5 ± 1.3	372	0.3 ± 1.0	166	0.8 ± 1.7	**<0.001**
Self-reported limited access to medical care due to the COVID-19 pandemic, yes	366 (68.8)	372	232 (62.4)	166	134 (80.7)	**<0.001**

Significant differences (*p* < 0.05) are in bold.

**Table 3:** Factors associated with cluster 2 (high scores of CSS-PL and SHAI) in binary logistic regression analysis.

	*B*	*SE*	*OR*	*95%CI*	*p*
Active working status, no	0.535	0.220	1.707	1.110–2.626	**0.015**
Chronic mental disorder, yes	0.933	0.286	2.542	1.452–4.450	**0.001**
The number of contacts with medical care units in the preceding month (without mental health services)	0.024	0.067	1.024	0.898–1.168	0.720
The number of contacts with mental health services in the preceding month	0.094	0.087	1.098	0.926–1.302	0.280
The use of online consultations with medical professionals in the preceding month, yes	0.342	0.232	1.408	0.893–2.218	0.141
Involvement in social meetings during the preceding month, yes	−0.278	0.203	0.757	0.509–1.127	0.170
Self-reported limited access to medical care due to the COVID-19 pandemic, yes	0.781	0.233	2.184	1.383–3.450	**0.001**

Significant associations (*p* < 0.05) are in bold.

## Data Availability

The data analyzed during this study are included in this published article. Further inquiries can be directed to the corresponding authors.
